# The Acute Effect of Oleic- or Linoleic Acid-Containing Meals on Appetite and Metabolic Markers; A Pilot Study in Overweight or Obese Individuals

**DOI:** 10.3390/nu10101376

**Published:** 2018-09-26

**Authors:** Shaan S. Naughton, Erik D. Hanson, Michael L. Mathai, Andrew J. McAinch

**Affiliations:** 1 Institute for Health and Sport, Victoria University, PO Box 14428, Melbourne, Victoria 8001, Australia; shaan.naughton@live.vu.edu.au (S.S.N.); edhanson@email.unc.edu (E.D.H.); micheal.mathai@vu.edu.au (M.L.M.); 2 The Florey Institute of Neuroscience and Mental Health, The University of Melbourne, 30 Royal Parade, Parkville, Melbourne, VIC 3052, Australia; 3 Australian Institute for Musculoskeletal Science (AIMSS), College of Health and Biomedicine, Victoria University, Melbourne, Victoria 8001, Australia

**Keywords:** dietary fats, appetite regulation, ghrelin, satiety, oleic acid, linoleic acid

## Abstract

Despite the abundance of plant-derived fats in our diet, their effects on appetite, and metabolic markers, remain unclear. This single-blinded 3-way cross-over pilot study aimed to investigate the ability of the two most abundant dietary plant-derived fats, oleic (OA) and linoleic (LA) acids, to modulate postprandial appetite and levels of circulating appetite and metabolic regulators in overweight/obese individuals. Meals were a high-carbohydrate control, a high-OA or a high-LA meal, and provided 30% of participants’ estimated energy requirements. Meals were consumed after an overnight fast, with blood samples collected over 3¼ h. Appetite parameters were assessed via a validated visual analogue scale questionnaire. Hormones and other circulating factors were quantified using multiplex immunoassays. Eight participants (age 45.8 ± 3.6 (years), body mass index 32.0 ± 1.3 (kg/m^2^)) completed the study. All meals significantly increased fullness and reduced desire to eat. The control and high-OA meals significantly decreased prospective food intake. The high-LA meal increased ghrelin levels (*p* < 0.05), a hormone which encourages food intake. This was coupled with a significant acute increase in resistin levels, which impairs insulin signaling. Taken together, this study indicates that in overweight/obese individuals, high-LA meals may promote excess energy intake and alter glucose handling, though a larger cohort may be required to strengthen results.

## 1. Introduction

Appetite is the main regulator of food intake, and as such is a key component in the regulation of energy intake and body weight [1]. Appetite is modulated by a range of endogenous substances produced by the stomach (ghrelin), small intestine (cholecystokinin, glucose-dependent insulinotropic peptide (GIP)), pancreas (insulin), adipose tissue (adiponectin, resistin) and brain (endocannabinoids), with many of these operating together, as well as in opposition [2]. Despite this, it is believed that when high-fat foods are available, as are found abundantly in current Western cultures, passive overconsumption occurs [3]. This is believed to be due to the body’s inability to gauge energy intake accurately, potentially leading to chronic excesses of energy intake, which may lead to weight gain and obesity [3]. Additionally, there is the possibility that our hormonal control of appetite, which evolved in conjunction with a low-fat diet, may be desensitized to overnutrition by modern high-fat dietary patterns [4,5]. With the exception of the first meal of the day, most meals are consumed by humans during the postprandial state [6]. As such, understanding how postprandial appetite regulation is influenced by our current nutritional environment is important to understand how our food intake affects subsequent intake and, ultimately, weight status.

When comparing the satiating effect of the macronutrients, lipids are the least satiating on an energy basis, possibly leading to excessive energy intake [7]. Despite this, the ability of specific fats to modulate appetite and food intake is yet to be fully elucidated. This study focuses on oleic acid (OA), a *n*-9 18 carbon monounsaturated fat, found primarily in olive oil, though also in other plant based oils, nuts, and vegetables, making it the most common dietary monounsaturated fat [8], and also on linoleic acid (LA) a *n*-6 18 carbon fatty acid, the most commonly consumed polyunsaturated fat, being found predominately in plant oils, nuts, meat, and animal products [9]. This is an important area of research, especially as the dietary availability of LA in the USA [9], Australia [10], Ireland [11], Barbados [12], Switzerland [13], Europe [14], and China [15], has increased dramatically (for example, over 2.5-fold in the USA [9]) in recent times. LA is the precursor of the two main endocannabinoids, anandamide and 2-arachidonyl glycerol, both of which promote hedonic food intake and adipose tissue energy storage [16]. Additionally, the endocannabinoid system is upregulated in obesity, which may further perpetuate excessive energy intake [17]. The importance of LA in the postprandial state is not only due to its potential to modulate hunger and satiety, it is also the precursor of arachidonic acid, which can be converted to several different inflammatory mediators [18]. As such, there is a possibility that a high intake of LA could perpetuate the low grade systemic inflammation found in overweight and obese individuals [19].

In comparison to LA, OA is not involved in the synthesis of inflammatory mediators, with several studies finding that OA and its endocannabinoid derivative oleoylethanolamide (OEA), reduce hunger and subsequent food intake [8,20,21]. Despite this, studies comparing OA- and LA-containing meals in an acute time frame have failed to find a difference in perception of hunger or appetite in the postprandial period [22,23].

The influence of OA or LA on blood glucose regulation and the development of diabetes is controversial and currently unclear [24,25,26,27]. Additionally, the role of these specific fatty acids (FA) in acutely modulating satiety-regulating hormones and perception of hunger and prospective food intake is yet to be fully elucidated, with no clear associations identified between them and appetite-modulating hormones [28]. Therefore, this pilot study aimed to assess the acute (2 h) response of appetite-regulating hormones, metabolic markers, and cytokines, as well as self-reported appetite parameters, following the consumption of mixed composition meals containing high levels of two of the most common dietary fats (LA or OA), in comparison to an energy-matched predominately carbohydrate-containing meal, in overweight or obese men and women.

## 2. Materials and Methods

### 2.1. Study Design

This study was designed as a single-blinded, 3-way cross-over pilot study. Due to the cost associated with measuring the circulating factors, many of which have not been investigated in response to acute FA intake, this pilot was undertaken to determine the efficacy of the protocol. With an aim of 10 subjects completing the study, the study had >80% power to discriminate a 1 standard deviation difference in plasma ghrelin levels between meals at significance level of *p* < 0.05. Ethical approval was obtained from the Victoria University human research ethics committee (HRETH 12/87), complying with the principles of the declaration of Helsinki. Participants were recruited May–August 2012 through flyers distributed on campus and via global email, with informed consent obtained from participants. The study took place in the University’s teaching clinic as part of a year-long research project. Each participant consumed the test meals after an overnight fast, with one meal consumed each week in a random order, with a minimum 5 day period between meals. Inclusion criteria for the study was a body mass index (BMI) >25 kg/m^2^ and a waist circumference >94 cm (male)/>80 cm (female), weight stable (no self-reported intentional or unintentional weight loss or gain over the preceding three months), and aged 18–60 years. Exclusion criteria were the self-reported presence of Type I or II diabetes mellitus, insulin resistance, heart, liver or kidney disease, pregnancy (or planning to become pregnant), breastfeeding, and the use of weight loss medication/supplementation or FA supplementation. For further details relating to the study design please see the CONSORT checklist in Table S1. Body composition (fat mass % and lean mass %) of fasted participants was measured using dual-energy x-ray absorptiometry (DXA) (Hologic Discovery QDR, Hologic (Australia) Pty Ltd., North Ryde, NSW, Australia) in the first week of the study. Anthropometric measurements were taken twice during the study and included height measurement, weight (Tanita HD-351 Digital Weight Scale, Tanita Pty. Ltd., Tokyo, Japan), waist circumference (measured at the narrowest point between the lower costal border and the iliac crest), hip circumference (taken at the level of the greatest posterior protuberance of the buttocks), blood pressure and heart rate (Omron 5 Series, Omron Healthcare, Inc., Palatine, IL, USA).

**Figure 1:**
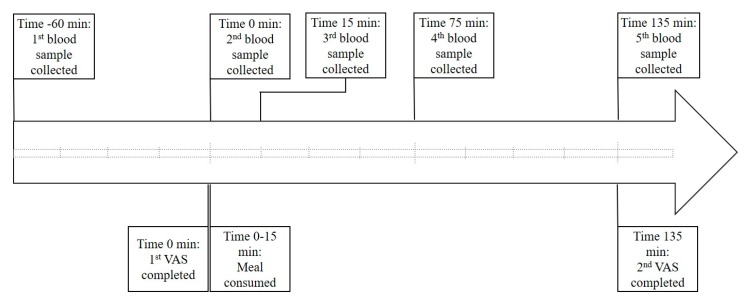
Meal challenge session timeline. Participants were required to consume the test meal in a 15 min time frame. Blood sampling occurred on arrival, 1 h later (immediately before consumption), immediately after consumption and then 1 and 2 h post consumption. Participants completed appetite assessment via validated visual analogue scale questionnaires (developed by Parker et al. 2004 [29]) immediately prior to consumption and 2 h after. min = minutes, VAS = visual analogue scale questionnaire. Each hatched line represents a 15 min interval.

Meal challenge sessions took place according to the timeline shown in Figure 1. Participants were instructed to consume a nutritionally balanced meal the night before testing and refrain from alcohol intake. Upon arrival, participants had a cannula inserted into their antecubital vein. All meals were consumed at the same time of day (between 9 a.m. and 10 a.m.) to control for diurnal hormonal variation, with a maximum of 15 min provided for consumption. Participants were encouraged to consume water during the sessions to maintain fluid levels and assist in the collection of blood, and instructed to refrain from exercise on the morning of test days.

### 2.2. Test Meal Compositions

The test meals were comprised of toasted bread, reduced sugar jam, icing sugar, and differing oil compositions (extra virgin olive oil, safflower oil, and coconut oil), resulting in control, high-OA (high-monounsaturated fatty acid (MUFA)), and high-LA (high polyunsaturated fatty acid (PUFA)) meals, with all ingredients used commonly found in Western diets. The meal compositions took into account palatability and the ability of participants to be blinded, with prior testing indicating that these criteria were met. This meal composition was chosen so as to replicate a prepared mixed composition meal that people would normally consume, as opposed to a liquid formula. As such, fiber-enriched bread was used, which also had a higher protein content than conventional white breads. Icing sugar was used to increase the energy density of the control meal to match those of the elevated-fat meals due to it homogenizing easily with the jam and also due to it not adding any further nutrients to the meal which would be absent in the elevated-fat meals. The high-fat meals had comparable ratios of all fats other than the one which had been elevated, with the amount of energy from the elevated fats in each high-fat meal also being matched as shown in Table 1. The two high-fat meals were isoenergetic and had matched contributions from macronutrients to energy content. Each test meal contained 13.74 ± 0.658 kJ/g as determined by nutritional analysis (performed using Xyris FoodWorks (Xyris Pty Ltd., Highgate Hill, QLD, Australia)). The meals were weighed to the nearest 0.1 gram and provided 30% of estimated daily energy intake, a figure based on the findings of large-scale studies indicating that breakfasts generally account for 20–25% of daily energy intake [30,31]. This was increased by 5% to account for the sessions running through to late morning, removing energy intake from foods generally consumed during this time period. Estimated energy intake was calculated using the Mifflin–St. Jeor equation which has been found to be the most accurate for overweight and obese individuals [32,33]. This equation took into account age, sex, height, and weight and was adjusted using appropriate activity factors based on participant-reported usual activity levels [34]. Hot water and lemon was provided with the meals to increase participant compliance and palatability, caffeinated beverages were not allowed while fasting or during the meal sessions due to caffeine’s influence on blood glucose levels [35].

### 2.3. Blood Collection and Analysis

A total of five blood samples were collected over each 3¼ h meal session, as shown in Figure 1. Blood glucose (average of triplicates) concentrations (all time points) were measured in serum samples using an auto-calibrating automated sampler (YSI 2300 Stat Plus Glucose L-Lactate analyzer, YSI Inc. Life Sciences, Yellow Springs, OH, USA). Levels of satiety and diabetes-related markers and cytokines (baseline, 1 h post meal, and 2 h post meal) were quantified using multiplex immunoassays (Bio-Plex Pro human diabetes 10-plex immunoassay (# 171A7001M) (measuring C-peptide, ghrelin, GIP, glucagon-like peptide 1 (GLP-1), glucagon, insulin, leptin, total plasminogen activator inhibitor-1 (PAI-1), resistin, and visfatin), Bio-Plex Precision Pro human cytokine 10-plex immunoassay (# 171A1001P) (measuring IL-1β, IL-2, IL-4, IL-5, IL-6, IL-10, IL-12 (p70), IL-13, interferon gamma (IFN-γ) and tumor necrosis factor alpha (TNF-α)) and Bio-Plex Pro human adiponectin single-plex immunoassay (# 171A7003M)) and a Bio-Plex^©^ 200 multiplex suspension array system (Bio-Rad Laboratories, Inc., Hercules, CA, USA), according to the manufacturer’s instructions.

### 2.4. Appetite Assessment

Participants completed an appetite questionnaire immediately before consumption of the meal and again after 2 h. The appetite questionnaire utilized a visual analogue scale (VAS) which has been found to be a reliable and valid tool for appetite assessment [36]. This comprised eight questions each followed by 100 mm horizontal lines, where 0 mm represented “sensation not felt at all” and 100 mm represented “sensation felt the greatest” with subjects asked to mark the line at the point which corresponded to how they were feeling at that particular time. The questionnaire developed by Parker et al. [29] has been used in similar studies assessing the effect of meal composition on appetite and gastrointestinal hormones in humans [37,38,39]. The distance from the beginning of the line to the participants’ mark was measured from the left-hand side to the nearest 0.5 mm.

### 2.5. Statistical Analysis

All statistical analysis was performed using Prism GraphPad version 7 (GraphPad Software Inc., La Jolla, CA, USA). For VAS scores a 2-way analysis of variance (ANOVA) was utilized. Difference between blood glucose levels in response to test meal consumption at each time point was determined via 2-way ANOVA with Tukey’s multiple comparisons test, as was change between time points within each test meal. For analysis of appetite-regulating compounds and inflammatory markers, area under the curve was utilized. This was calculated as the net change from baseline to account for negative peaks, i.e., reductions in circulating concentrations over the period of sampling. Analytes that did not have a full set of data inside the reporting range were excluded from analysis.

## 3. Results

Eight participants completed the study, 5 females and 3 males. For further information regarding participant recruitment and numbers please see Figure S1. The average age was 45.8 ± 3.6 years and average BMI 32.0 ± 1.3 kg/m^2^. Further participant characteristics are shown in Table 2.

**Figure 2:**
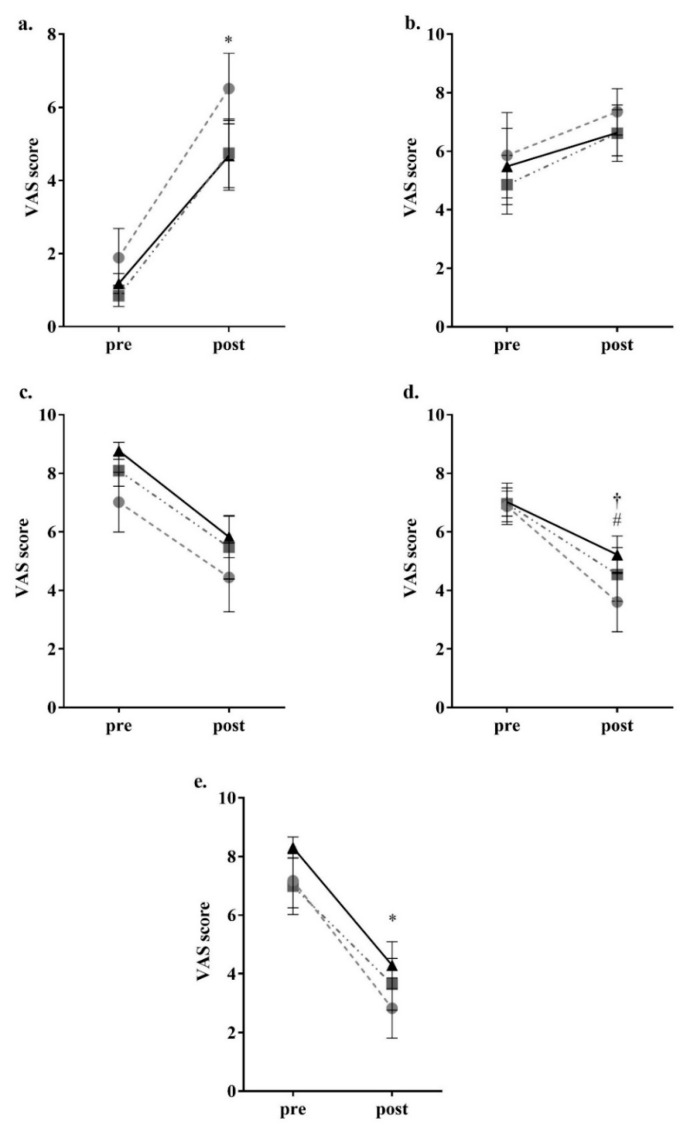
Participant visual analogue scale appetite parameter responses. (**a**) Fullness; (**b**) Satisfaction; (**c**) Desire to eat; (**d**) Prospective food intake; (**e**) Hunger. ● = control meal, ■ = high-OA, ▲ = high-LA. Assessed through completion of validated visual analogue questionnaires and measured to the nearest 0.5 mm. Pre = VAS (visual analogue scale questionnaire) completed 15 min prior to consumption; Post = VAS completed 2 h post consumption. * = *p* < 0.05 all meals comparing pre and post consumption. † = *p* < 0.05 for control meal comparing pre and post consumption. # = *p* < 0.05 for high-OA comparing pre and post consumption. All data shown as mean ± SEM, all data points *n* = 8.

**Figure 3:**
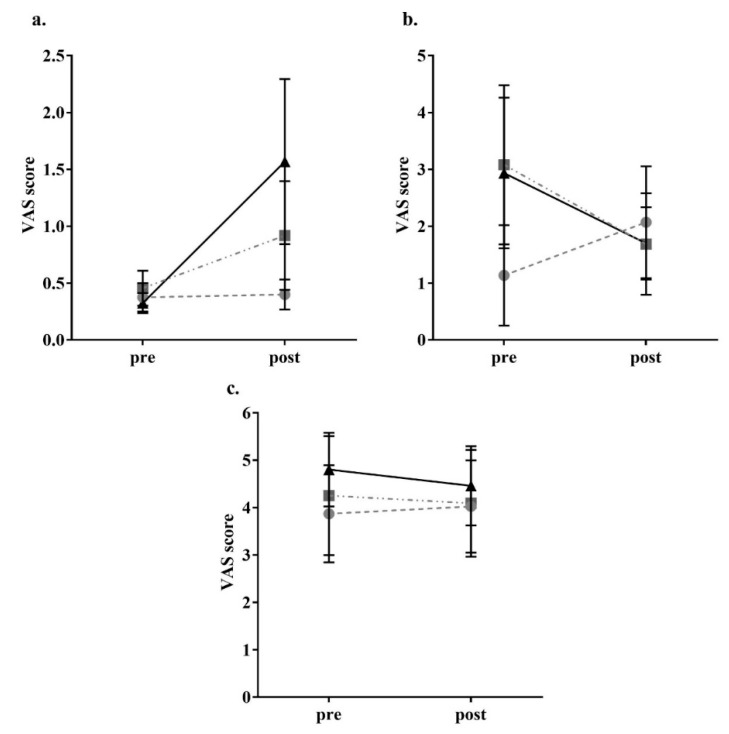
Participant visual analogue scale other sensations responses. (**a**) Anxiety; (**b**) Nausea; (**c**) Fatigue. ● = control meal, ■ = high-OA, ▲ = high-LA. Assessed through completion of validated visual analogue questionnaires and measured to the nearest 0.5 mm. Pre = VAS (visual analogue scale questionnaire) completed 15 min prior to consumption; Post = VAS completed 2 h post consumption. All data shown as mean ± SEM, all data points *n* = 8.

For the parameters related to appetite, there was a significant reduction in feeling of hunger (control *p* < 0.05, high-OA *p* < 0.05, high-LA *p* < 0.05) and a significant increase in feeling of fullness (control *p* < 0.05, high-OA *p* < 0.05, high-LA *p* < 0.05) after consumption of all meals as shown in Figure 2. There was no significant change to perceived satisfaction and desire to eat following consumption of all three meals. In regard to prospective food intake amount (with ‘none’ being the bottom of the scale and ‘A large amount’ being the top of scale), there was a significant decrease following consumption of the control and high-OA meals control *p* < 0.05, high-OA *p* < 0.05), though not following consumption of the high-LA meal (*p* = 0.183), as shown in Figure 2. VAS results showed no changes to perception of fatigue, nausea, or anxiety (though the high-LA meal elicited a nonsignificant trend to increase anxiety *p* = 0.095) as a result of the consumption of any of the test meals, as shown in Figure 3, thus it is unlikely that these variables influenced perceived hunger as a result of consumption of the different test meals. 

**Figure 4:**
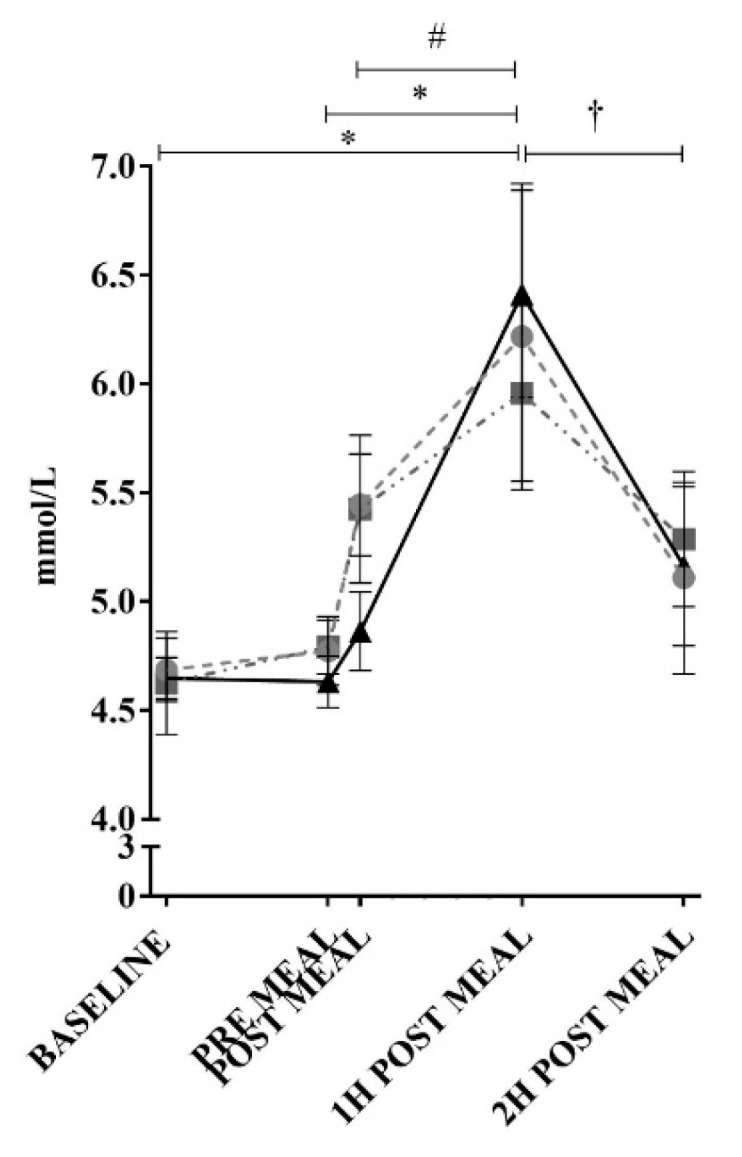
Blood glucose responses to ingestion of test meals. ● = control meal, ■ = high-OA, ▲ = high-LA. Measured in plasma using an auto-calibrating automated sampler. * = *p* < 0.05 all meals comparing time points. † = *p* < 0.05 for control and high-LA comparing time points. # = *p* < 0.05 for high-LA comparing time points. All data shown as mean ± SEM, all data points *n* = 8 (average of triplicates).

Blood glucose measurement showed no significant difference between the test meals at any of the time points. There was a significant increase in blood glucose concentrations following the consumption of all test meals between baseline and 1 h post meal consumption (control *p* < 0.05, high-OA *p* < 0.05, high-LA *p* < 0.05) and between pre-meal and 1 h post consumption (control *p* < 0.05, high-OA *p* < 0.05, high-LA *p* < 0.05), as shown in Figure 4.

When comparing between time points for the high-LA meal, the blood glucose concentration at the 1 h time point was significantly increased (*p* < 0.05) compared to immediately post consumption; however, none of the other meals were increased from immediately post consumption to 1 h post consumption. There was a significant decrease in blood glucose concentrations between 1 and 2 h post consumption for the control and high-LA meals (control *p* < 0.05, high-LA *p* < 0.05), though not for the high-OA meal. Despite these alterations, calculation of area under the curve (AUC) of the blood glucose levels in the 2 h post consumption of each test meal found no significant differences in blood glucose response between the different test meals (data not shown, Control AUC = 86.24 ± 5.25 AU, high-OA AUC = 85.21 ± 3.52 AU, high-LA AUC = 85.08 ± 3.53 AU (mean ± SEM)).

**Figure 5:**
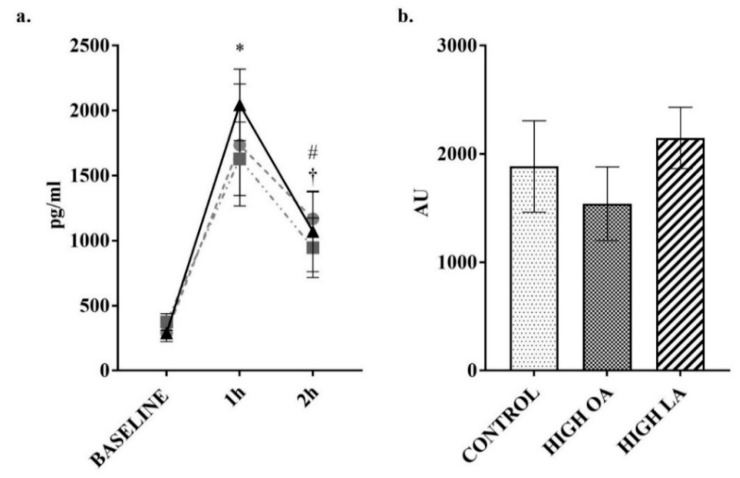
Change in insulin over 2 h postprandial period. (**a**) plotted values. ● = control meal, ■ = high-OA, ▲ = high-LA; (**b**) Net area under curve (AUC) from baseline. Measured in plasma using a multiplex immunoassay. All data displayed as mean ± SEM, all data points *n* = 8. * = significantly different (*p* < 0.05) to baseline for all test meals. # = significantly different (*p* < 0.05) to baseline for control meal. † = significantly different (*p* < 0.05) to 1 h for the high-LA test meal.

Changes to insulin concentrations were measured as previous research indicates the potential modulation of postprandial responses by monounsaturated fats [40]. Consumption of all test meals resulted in a significant increase in insulin at the 1 h post consumption time point (control *p* < 0.05, high-OA *p* < 0.05, high-LA *p* < 0.05), as shown in Figure 5a. In response to consumption of the control meal, insulin levels were still significantly elevated compared to baseline at the 2 h post consumption time point (*p* < 0.05). In response to the consumption of the high-LA test meal, there was a significant decrease (*p* < 0.05) in insulin concentrations between the 1 and 2 h post consumption time points. Despite this, no significant differences were found between the test meals during subsequent net AUC analysis, as can be seen in Figure 5b. 

**Figure 6:**
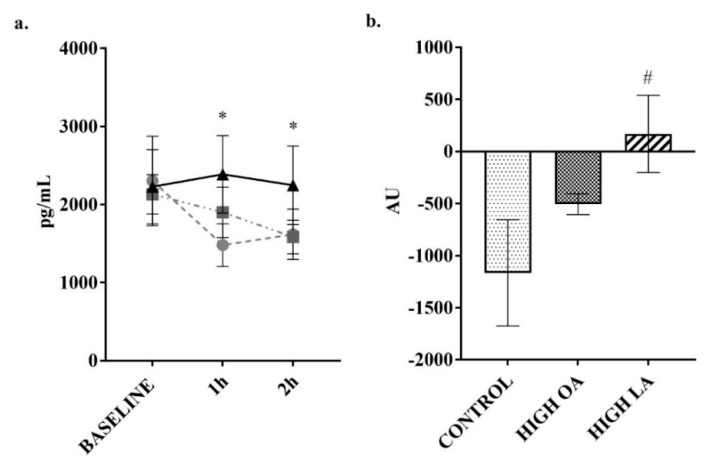
Change in ghrelin over 2 h postprandial period. (**a**) plotted values, ● = control meal, ■ = high-OA, ▲ = high-LA; (**b**) Net AUC from baseline. Measured in plasma using a multiplex immunoassay. All data displayed as mean ± SEM, all data points *n* = 8. * = significantly different (*p* < 0.05) at this time point compared to baseline for control meal. # = significantly different (*p* < 0.05) to control meal.

Ghrelin was a hormone of interest in this study due to previous research indicating that postprandial levels can be modulated by the level of FA saturation [28]. In response to the high-LA test meals’ consumption, there was a significant increase (*p* < 0.05) in ghrelin production from baseline, compared to the control meal when analyzing the net AUC. Additionally, there was a significant reduction in ghrelin concentrations following consumption of the control meal at both the 1 (*p* < 0.05) and 2 h (*p* < 0.05) time points when compared to baseline as shown in Figure 6a.

**Figure 7:**
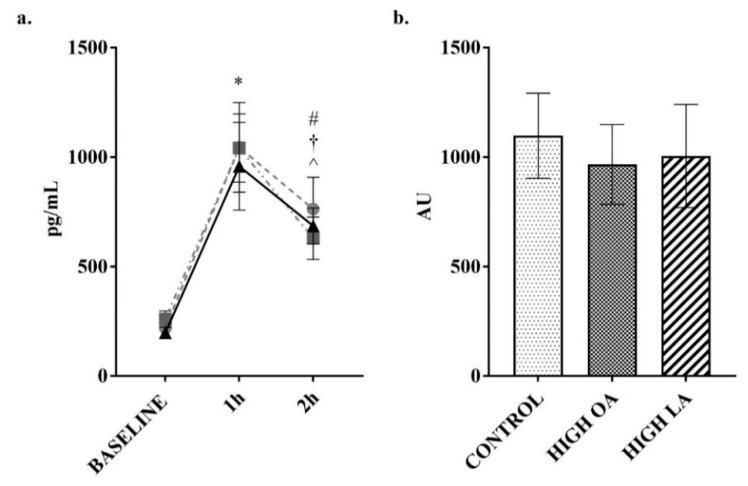
Change in glucose-dependent insulinotropic peptide (GIP) over 2 h postprandial period. (**a**) plotted values, ● = control meal, ■ = high-OA, ▲ = high-LA; (**b**) Net AUC from baseline. Measured in plasma using a multiplex immunoassay. All data displayed as mean ± SEM, all data points *n* = 8. * = significantly different to baseline for all test meals. # = significantly different (*p* < 0.05) to baseline for control, † = significantly different (*p* < 0.05) to baseline for high-LA test meal. ^ = significantly different (*p* < 0.05) to 1 h for the high-OA test meal.

**Figure 8:**
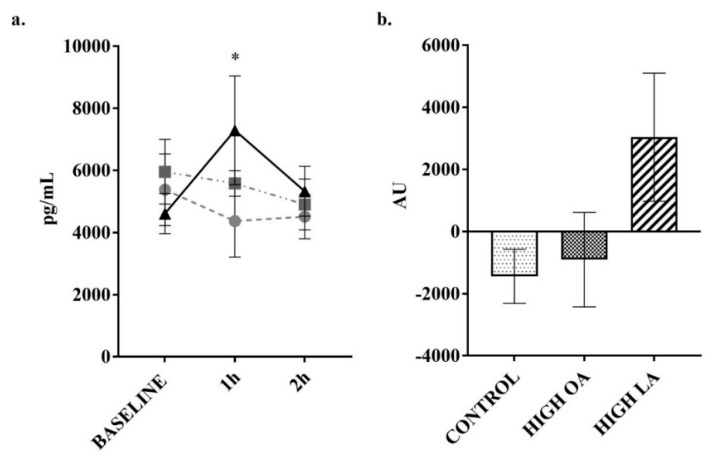
Change in resistin over 2 h postprandial period. (**a**) plotted values, ● = control meal, ■ = high-OA, ▲ = high-LA; (**b**) Net AUC from baseline. Measured in plasma using a multiplex immunoassay. All data displayed as mean ± SEM, all data points *n* = 8. *, significantly different (*p* < 0.05) at this time point compared to baseline for the LA meal.

The plasma concentration of the hormone GIP can be altered acutely by food intake [41]. In response to consumption of all test meals there was a significant increase in GIP at the 1 h post consumption time point (all *p* < 0.05), as shown in Figure 7a. Consumption of the control (*p* < 0.05) and LA (*p* < 0.05) test meals resulted in a sustained significant difference when compared to baseline at the 2 h post consumption time point, though not for the high-OA test meal. At the 2 h time point there was a significant decrease in GIP concentrations following consumption of the high-OA test meal (*p* < 0.05), though there were no significant differences in net AUC between the different meals. At 1 h post meal consumption, there was a significant increase (*p* < 0.05) in resistin following consumption of the high-LA test meal, though this failed to result in a significant difference in net AUC for the test period (*p* = 0.13 compared to control), as shown in Figure 8b.

**Figure 9:**
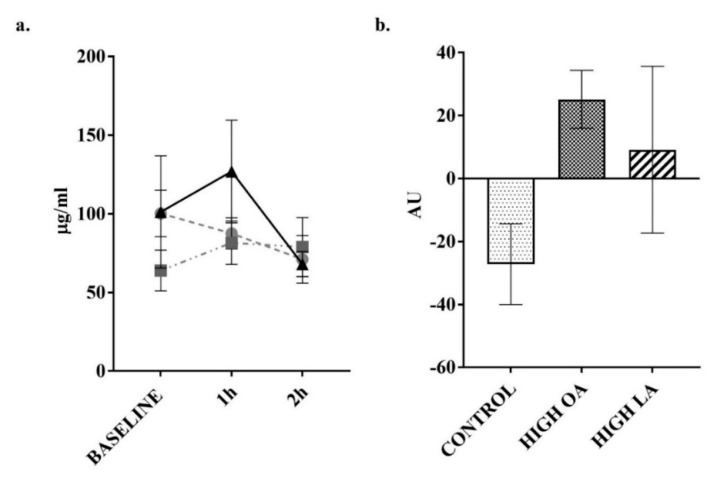
Change in adiponectin over 2 h postprandial period. (**a**) plotted values, ● = control meal, ■ = high-OA, ▲ = high-LA; (**b**) Net AUC from baseline. Measured in plasma using a multiplex immunoassay. All data displayed as mean ± SEM, all data points *n* = 8.

As shown in Figure 9, there were no significant changes in adiponectin concentrations or adiponectin net AUC following consumption of any of the test meals.

There was no significant change to GLP-1, glucagon, leptin, IL-1β, IL-6, IL-10, IL-13, TNF-α, or C peptide in response to test meal consumption (see Figures S2–S10). Measurement of PAI-1, visfatin, IFN-γ, IL-2, IL-3, IL-4, IL-5, and IL-12p70 failed to yield complete data sets due to samples being outside the range of determination, and as such were removed from statistical analysis. 

There were no significant differences observed between baseline measurements (pre-test meal consumption) for any of the analytes or appetite parameters measured. No important harms or unintended effects were observed.

## 4. Discussion

The modern food environment has an abundance of cheap, highly palatable plant-derived oils, yet the ability of different FA to modulate appetite and food intake is currently unclear, particularly in overweight and obese individuals. In this study, using carefully designed meals with matched energy from macronutrients and matched contents of the specifically elevated fatty acids in the two high-fat meals, we were able to show regulation of postprandial appetite markers and perceived appetite by either OA or LA.

The participants were all overweight or obese and had elevated central adiposity, though their fasting blood glucose levels indicate they had normal blood glucose management when compared to national guidelines, and were normotensive [42,43]. Though blood glucose responses showed no significant differences between test meals, there was a significant increase in blood glucose concentrations between immediately post consumption and the 1 h time point for the LA meal. Additionally, there was a significant decrease in blood glucose concentrations between the 1 and 2 h postprandial time points in response to consumption of the control and LA test meals, which was not seen following consumption of the OA meal. Taken together, this indicates that the LA meal resulted in a faster peak and then decline in blood glucose concentrations. As the two high-fat test meals had matched fat content, this difference in blood glucose responses cannot be attributed to postprandial lipidaemia or the FA chain length, as both were 18 carbon chains. Moreover, the intestinal absorption of OA and LA is comparable (99.8–99.9%), indicating that malabsorption is unlikely to be a contributing factor [44]. A meta-analysis comparing low- and high-MUFA diets found that a high MUFA intake correlated with a decrease in HbA1c in diabetic individuals [45], with another study finding that a high-MUFA diet decreases fasting blood glucose when compared to high-PUFA diets [46]. Additionally, studies have found that in the long term, high-OA diets improve insulin sensitivity and are capable of lowering fasting insulin [47], and decreasing the postprandial insulin response when compared to a high-LA meal [48], though as found here, there were no significant differences in postprandial insulin responses between the test meals. In regard to the control meal, the insulin levels were still significantly elevated at the 2 h time point when compared to baseline, most likely due to the higher carbohydrate content of this meal.

Blood glucose responses in this study may have also been affected by resistin production, with a significant increase at the 1 h time point following consumption of the high-LA test meal, with a trend (*p* = 0.055) towards a difference in net AUC across the testing period when compared to the control meal. As resistin is an adipokine which has been shown to interfere with intracellular insulin signalling, and also stimulates monocyte and macrophage inflammatory cytokine production, this may contribute to the systemic low-grade inflammation observed in obesity [49,50]. Treatment of cultured 3T3-L1 adipocytes with OA has found that when compared to the trans isomer of OA (elaidic acid) there is a significant reduction in resistin expression [51], though another group has found that arachidonic acid (AA) is capable of decreasing resistin expression in the same cell type [52]. While human and mouse resistin only share a 59% homology, and it currently appears there are differences in regulation and function, human resistin is involved in inflammation-induced insulin resistance in the liver and skeletal muscle and may also be implicated in cardiovascular disease [53], though the influence of obesity on resistin levels is unclear [54,55,56]. Despite this evidence, there appears to be an absence of studies comparing the effect of dietary OA or LA on resistin levels, though one large-scale study (~6600 participants) has found that adherence to the Mediterranean diet and MUFA intake is associated with lower resistin concentrations [57]. Moreover, it appears that the role of LA in modulating postprandial resistin levels in humans has not been sufficiently investigated to draw further inference. 

Adiponectin is also an adipokine capable of influencing blood glucose levels, though contrary to resistin, it increases the sensitivity of tissues, such as skeletal muscle, to insulin, resulting in greater glucose uptake [58]. Interestingly, treatment of both cultured human and mouse adipocytes with OA has been found to reverse TNF-α induced depletion of adiponectin mRNA expression, on its own, and in combination with the antioxidant phenol hydroxytyrosol, commonly found in olive oil [59]. This is supported by studies showing an increase in circulating adiponectin in a subsection of the Nurse’s Study cohort who habitually consumed a Mediterranean diet [60], in a group of overweight men prescribed a Mediterranean style diet [61], and in a mixed-sex study that aimed to increase monounsaturated fats to 10% or more of energy consumed [62]. Despite this, the current study did not find significant differences in adiponectin levels following meal consumption in an acute setting.

This study also found that consumption of the high-LA test meal resulted in an increase in ghrelin production when the net AUC was compared to the response to the control meal. Ghrelin is a neuropeptide which promotes food intake, with circulating levels generally increasing prior to food intake and dropping in the postprandial period, generally decreasing within 1 h of food consumption [63,64]. This indicates that LA may be able to modulate the normal ghrelin response to feeding. This response to ghrelin is in contrast to what was found by Stevenson et al. [65], in lean individuals following consumption of a 35% fat meal (with 21% from PUFA) which elicited no postprandial change to ghrelin when compared to a high-carbohydrate control meal. This may indicate that the higher amount of fat (and load of PUFA) consumed in the current study (55% of energy from fat, of which 70% was LA) may have influenced the results. There is also the possibility that the increased adiposity of the subjects (which has been found to correlate with lower ghrelin production [66]) in the present study or the length of postprandial period (2 vs. 4 h in Stevenson Paton, and Cooper (2017)) may account for this variation between the two studies. Adding to this increase in ghrelin, analysis of VAS responses to perceived prospective food intake found that the perceived amount of food which participants believed they could comfortably consume decreased post consumption for both the control and high-OA, but not the high-LA meals. This indicates that though hunger had decreased and fullness increased, there was not a decrease in how much they felt they could eat. This possible indication of the promotion of hedonic food intake can be stimulated by ghrelin, which was increased following consumption of the LA meal in the current study. This is an important factor when considering modern environments of abundant, high-fat, and especially high-LA, palatable foods [67,68].

GIP was increased at 1 h compared to baseline following consumption of all meals, which was sustained at the 2-h time point for the control and LA meals, though there were no significant differences in net AUC between meals. GIP secretion in response to both OA and LA consumption has been demonstrated previously, though over a considerably longer period of time (30 h) with incremental fat consumption [69]. Despite this, other research comparing MUFA-, PUFA- and saturated fatty acid (SFA)-containing meals in people with metabolic syndrome over a 6 h period found no significant difference in AUC between the PUFA (LA) and MUFA (OA) meals when analysing AUC [70]. In agreement with this, earlier research has found no difference in 3 h responses to ingestion of high-LA and -OA amounts at any measured time points [71], indicating that the level of saturation may not affect the GIP response, as was found in the present study. Additionally, changes to GLP-1 concentrations were not observed in the present study. This is not surprising due to the requirement of stimulation of L-type enterocytes in the ileum and colon to trigger secretion of GLP-1, with the time course of this study not allowing sufficient time for progression to this area of the gastrointestinal tract [72].

The finding of no significant difference in perceived appetite between the test meals following consumption is in line with a study conducted in lean healthy men consuming high-OA and -LA breakfasts [23]. This study failed to elicit differences in perceived appetite following a mixed-composition breakfast, nor was there a difference in energy intake at an ad libitum buffet style lunch following the meal intake, though this study did not look at hormonal responses [23]. Interestingly, in a study using 15 healthy normal weight mixed-gender participants, energy intake at a subsequent meal and over the following 24 h was reduced following consumption of both extra virgin olive oil and high-OA sunflower oil-containing meals, compared to a high-LA meal, with a corresponding increase in circulating OEA [73]. Despite this, a number of other studies have also found no significant difference between test meals for any appetite parameters measured when comparing OA and LA (and in some instances SFA) meals in an acute timeframe [22,74,75] or when the fats are delivered via ileal infusion [76], which may show an influence of gender or weight status. This may indicate that measurement of hormones involved in appetite modulation may be a better indicator of how appetite responses are affected by food intake, than self-reporting.

Though this was only a pilot study, the likelihood of significant results would most likely have been increased with a larger cohort of participants, especially as the number of participants who completed the study was below the number calculated to achieve adequate power, and would address the issue of type II errors. Assuming equal variance in results with an expanded cohort, a sample of 23 would be required to determine differences between ghrelin concentrations following consumption of the OA and LA meals and samples of 30 and 38 to determine differences in resistin and insulin respectively. Additionally, expansion of the cohort would allow for further testing of ordering effects. The random-ordered three-way cross-over study design helped to remove the variable of menstrual cycles modulating hormone levels in the female participants. The reliance on self-reported exercise levels to determine energy requirements is a limitation of the study, though as the same amount of energy was provided to participants in each meal, this is not of overt concern. The careful meal design was a strength of the study, with matched energy contents from different fats being able to be achieved through careful manipulation of ingredient quantities.

## 5. Conclusions

The results of this pilot study show that OA and LA have the potential to modulate postprandial hormonal responses in overweight and obese individuals. With a high-LA meal leading to an increase in ghrelin, a key hormone in promoting food intake, potentially evidenced by this meal being the only one tested to not elicit a decrease in prospective food intake in the postprandial period. Consumption of the LA meal also caused a spike (1 h post meal consumption) in the levels of resistin, an adipokine which decreases insulin sensitivity. Additionally, consumption of the high-LA meal caused a significant peak in blood glucose levels 1 h post consumption which was not observed following the same load of OA, indicating potential changes to glucose management. Moreover, this study is the first of its kind (to the author’s knowledge) to investigate such a wide range of appetite-modulating hormones in response to an acute fatty acid intake with matched loads. Expansion of the cohort would enable further investigation of the role of LA in modulating resistin and ghrelin levels in overweight and obese individuals, and may provide insight into how dietary fats are capable of affecting postprandial appetite and metabolism. As ghrelin has a role in hedonic food intake, the assessment of ad libitum energy intake at a subsequent meal may also be of interest.

## Figures and Tables

**Table 1:** Nutritional composition of experimental meals per 100 g.

Nutrient	Control	High-OA	High-LA
Carbohydrate (g)	50.9	33.7	33.7
Carbohydrate (% E)	73.46	39.74	39.74
Protein (g)	4.8	5.8	5.8
Protein (% E)	6.91	6.84	6.84
Fat (g)	7.3	21.5	21.5
Fat (% E)	23.12	55.13	55.13
Saturated (g)	2.5	3.1	3.1
MUFA (g)	2.5	15.1	3.1
Oleic Acid (g)	2.4	14.8	2.9
PUFA (g)	2.3	3.2	15.2
Linoleic Acid (g)	2.2	3.0	14.9
Fiber (g)	3.2	4.0	4.0
Omega 6:Omega 3	16.7	13.1	77.2

OA; Oleic acid, LA; Linoleic acid, % E; percentage of total energy; MUFA; monosaturated fatty acid, PUFA; polyunsaturated fatty acid.

**Table 2:** General participant characteristics.

Parameter		Average ± SEM
Age (years)		45.8 ± 3.6
Height (cm)		169.5 ± 2.2
Weight (kg)		92.2 ± 4.5
BMI (kg/m^2^)		32.0 ± 1.3
Waist Circumference (cm)	Female	102.4 ± 7.1
	Male	108.6 ± 7.9
Hip Circumference (cm)	Female	119.6 ± 4.1
	Male	111.3 ± 5.7
Waist-To-Hip Ratio	Female	0.83 ± 0.03
	Male	0.97 ± 0.01
Fat Mass (% of Total Mass)		37.4 ± 2.9
Lean Mass (% of Total Mass)		55.7 ± 2.8
Systolic Blood Pressure (mmHg)		128.4 ± 4.1
Diastolic Blood Pressure (mmHg)		82.3 ± 3.5
Heart Rate (bpm)		73 ± 4.4
Fasting Serum Glucose (mmol/L)		4.6 ± 0.1

BMI; body mass index, SEM; standard error of the mean.

## Supplementary Materials

The following are available online at http://www.mdpi.com/2072-6643/10/10/1376/s1, Figure S1: CONSORT diagram, Figure S2: Change in glucagon over 2 h postprandial period, Figure S3: Change in leptin over 2 h postprandial period, Figure S4: Change in GLP-1 over 2 h postprandial period, Figure S5: Change in IL-β over 2 h postprandial period, Figure S6: Change in IL-6 over 2 h postprandial period, Figure S7: Change in IL-10 over 2 h postprandial period, Figure S8: Change in IL-13 over 2 h postprandial period, Figure S9: Change in C peptide over 2 h postprandial period, Figure S10: Change in TNF-α over 2 h postprandial period, Table S1: CONSORT checklist.
